# Genetic Etiology of Developmental and Epileptic Encephalopathy in a Turkish Cohort: A Single-Center Study with Targeted Gene Panel and Whole Exome Sequencing

**DOI:** 10.3390/genes16101152

**Published:** 2025-09-28

**Authors:** Deniz Sunnetci-Akkoyunlu, Bulent Kara, Tolgahan Ozer, Adnan Deniz, Ayfer Sakarya-Gunes, Elif Busra Isik, Buket Dogruoglu, Zeynep Ilkay, Mehtap Yilmaz, Sumeyye Sahin, Seda Eren-Keskin, Naci Cine, Hakan Savli

**Affiliations:** 1 Department of Medical Genetics, Faculty of Medicine, Kocaeli University, 41001 Kocaeli, Türkiye; tolgahan.ozer@kocaeli.edu.tr (T.O.);; 2 Department of Pediatric Neurology, Faculty of Medicine, Kocaeli University, 41001 Kocaeli, Türkiye; bulent.kara@kocaeli.edu.tr (B.K.); ayfer.gunes@kocaeli.edu.tr (A.S.-G.); 3 Department of Cardiac Surgery, University Hospital Erlangen, 91054 Erlangen, Germany

**Keywords:** epileptic encephalopathy, whole exome sequencing, *PIEZO2*, *TSC1*, *CTBP1*, *NALCN*, *TSC2*

## Abstract

Background: Developmental and Epileptic Encephalopathy (DEE) is a severe and heterogeneous neurological disorder in infancy/early childhood. DEE’s genetic and phenotypic variability complicates diagnosis and treatment. This retrospective study aimed to identify genetic variants and explore genotype–phenotype correlations in children with DEE using a targeted epilepsy gene panel (TGP) and Whole Exome Sequencing (WES). Patients and Methods: Medical records of children who underwent custom-designed 55-gene TGP and WES were reviewed. The diagnostic yield of each method was determined based on the detection of pathogenic (P) and likely pathogenic (LP) variants. Results: A total of 129 patients (66 males, 63 females) underwent TGP, which identified P/LP variants in 29 cases (22.48%). Variants were detected in *SCN1A*, *KCNQ2*, *STXBP1*, *CDKL5*, *PCDH19*, *PLCB1*, *WWOX*, *SCN2A*, *FGF12*, *HCN1*, *SCN8A*, and *SLC35A2*. WES further identified several variants in children with West syndrome. A *TSC1* variant was detected in a patient without cutaneous stigmata of tuberous sclerosis complex. The *NALCN* variant in a patient was linked to Infantile Hypotonia with Psychomotor Retardation and Characteristic Facies 1. A *CTBP1* variant associated with extremely rare Hypotonia, Ataxia, Developmental Delay, and Tooth Enamel Defect Syndrome was detected in another patient. A *PIEZO2* variant—associated with Marden–Walker syndrome—was found in a child with Early Infantile Developmental and Epileptic Encephalopathy. Conclusions: These findings highlight the extensive genetic heterogeneity and phenotypic variability of DEE. WES demonstrates substantial value in identifying novel gene-disease associations and may be considered as a first-tier diagnostic tool in epilepsy and DEE.

## 1. Introduction

Epileptic encephalopathy is a severe neurological disorder characterized by intractable seizures and severe cognitive and behavioral impairments that can be seen at any age and can progressively worsen over time [1]. According to the International League Against Epilepsy (ILAE), the term “Developmental and Epileptic Encephalopathy” (DEE) describes epileptic syndromes marked with developmental regression independent of seizure activity and having a genetic etiology [2]. Identifying the genetic etiology responsible for DEE is crucial for accurate diagnosis, prognosis, and the management of targeted treatment strategies [3].

With the advances in genomic technologies, particularly in Next Generation Sequencing (NGS) methods, the diagnostic process has been revolutionized and has elucidated the underlying genetic etiology. The current Online Catalog of Human Genes and Genetic Disorders (OMIM) includes 116 DEE-related genes (https://omim.org/phenotypicSeries/PS308350, accesed on 16 August 2025).

Recent NGS-based studies have further illuminated the extensive genotypic and phenotypic heterogeneity among DEE syndromes such as Early Infantile Developmental and Epileptic Encephalopathy (EIDEE), West Syndrome (WS), and Dravet Syndrome (DS). For example, in a large Chinese cohort of over 2200 children with early-onset epilepsies, including Lennox–Gastaut, WSs, and DSs, the authors reported that while certain genes yield predictable phenotypes (e.g., *SCN1A* in DS), others show broad variability depending on variant type and modifier effects [4]. Another recent scoping review of studies in infants with DEE identified *STXBP1*, *SLC1A2*, *CDKL5*, *SCN1A*, and *KCNT1* as frequently mutated genes; each was associated with distinctive electroclinical features (age at seizure onset, seizure type, EEG pattern, degree of developmental delay) though with substantial inter-individual variation [5].

In EIDEE cohorts, different genes like *SCN2A*, *KCNQ2*, and *SCN1A* produce overlapping phenotypes, but the specific variant class (truncating vs. missense), inheritance (de novo vs. inherited), and even variant location (e.g., domain within the protein) substantially modulate age at onset, seizure severity, response to ASMs, and developmental outcomes [6].

With respect to WS, mutations in *CDKL5* are often associated with very early onset epileptic spasms, profound developmental impairment, and often atypical EEG features; yet other WS cases with different genes show more variable developmental trajectories and responses to treatment [5].

In DS, the genotype–phenotype correlations are somewhat more consistent but still show heterogeneity. In a large international cohort (~1000+ *SCN1A* mutation carriers), missense variants in conserved or functionally critical regions are strongly associated with classic DS (earlier onset, severe febrile seizures, more frequent status epilepticus), whereas variants in less conserved regions or truncating mutations at certain gene ends may be more often linked with milder phenotypes (e.g., GEFS+) [7]. Also, cases of co-occurring pathogenic variants (e.g., *SCN1A*+*SCN2A*) have been shown to exacerbate severity: earlier seizure onset, more types of seizures, poorer developmental outcomes [8].

These findings underscore that for DEE syndromes, knowing which gene is mutated is critical, but it is often only part of the story: variant type, location, inheritance pattern, interacting/modifier genes, and even environmental or therapeutic modifiers also influence clinical presentation and prognosis.

Targeted gene panels offer several advantages over wide-ranging approaches such as Whole-Exome Sequencing (WES) or Whole-Genome Sequencing (WGS) [9]. These panels focus on a curated set of genes with established links to DEE, thereby reducing the complexity of data analysis and interpretation, providing higher coverage and depth for the selected genes, reducing costs and turnaround times [10]. Previous studies have reported custom-designed epilepsy-related gene panels with diagnostic yields, which range from 18 to 46.2% [3,10,11,12,13,14,15,16,17]. However, gene panels have inherent limitations due to their static nature; as the field of epilepsy genetics evolves rapidly, novel causative genes may not be included in existing panels, potentially leading to missed diagnoses. Despite these challenges, the complementary nature of WES and gene panels underscores their utility in clinical practice. While WES offers a broader diagnostic scope, integrating these two approaches could further enhance diagnostic accuracy.

In this retrospective study, investigation of the genetic etiology of DEE in a cohort of 129 children using a combination of TGP and WES was the aim. Genotype–phenotype correlations were explored to better understand the relationship between genetic variants and clinical presentations.

## 2. Patients and Methods

### 2.1. Patients

In this single-center retrospective cohort study, records of children who underwent NGS testing at the Department of Medical Genetics between October 2017 and July 2020 were retrieved. Inclusion criteria encompassed patients with clinical symptoms suggestive of DEE, such as epilepsy and developmental delay, occurring in infancy and early childhood. Notably, patients whose clinical findings were explained by one of the genetic testing methods, such as conventional karyotyping and molecular karyotyping, were excluded from the study. 129 patients who met the criteria were included in the study. This study was conducted in accordance with the principles of the Declaration of Helsinki and approved by the Ethics Committee of Kocaeli University, under the protocol number 2023/367. Informed consent was obtained from legal guardians.

### 2.2. Targeted Gene Panel and Whole Exome Sequencing

A custom-designed targeted gene panel included 55 genes known to be implicated in various forms of DEE based on current literature and databases such as OMIM: *ARX*, *CDKL5*, *SLC25A22*, *STXBP1*, *SPTAN1*, *SCN1A*, *SCN9A*, *KCNQ2*, *ARHGEF9*, *PCDH19*, *PNKP*, *SCN2A*, *PLCB1*, *SCN8A*, *KCNT1*, *ST3GAL3*, *TBC1D24*, *GNAO1*, *SZT2*, *GABRA1*, *PIGA*, *NECAP1*, *SLC35A2*, *DOCK7*, *HCN1*, *SLC13A5*, *KCNB1*, *GRIN2B*, *WWOX*, *AARS*, *SIK1*, *DNM1*, *KCNA2*, *EEF1A2*, *SLC12A5*, *ITPA*, *ALG13*, *FRRS1L*, *ARV1*, *SLC25A12*, *GUF1*, *SLC1A2*, *CACNA1A*, *GABRB3*, *UBA5*, *GABRB1*, *GRIN2D*, *FGF12*, *AP3B2*, *DENND5A*, *CAD*, *MDH2*, *SCN1B*, *SYNJ1*, *HNRNPU*.

The primers were designed with the Ion AmpliSeq Designer tool v1.22 (Life Technologies, http://www.ampliseq.com, accesed on 16 August 2025). The final assay design consisted of a total of 1327 amplicons.

Genomic deoxyribonucleic acid (DNA) was extracted from peripheral blood using EZ1 DNA Blood 200 μL Kit (Qiagen, Hilden, Germany). Amplicon-based sequencing libraries were prepared using the Ion AmpliSeq library 2.0 kit and custom primers following the manufacturer’s instructions (Life Technologies, Delhi, India). Sequencing was performed on an Ion Torrent S5 sequencer using an Ion 520 chip according to the manufacturer’s instructions (Life Technologies, Delhi, India).

WES was performed on the NovaSeq 6000 (Illumina, San Diego, CA, USA) after library preparation via QIAseq Human Exome Kit (Qiagen, Hilden, Germany) according to the manufacturer’s instructions.

### 2.3. Variant Evaluation

Reads were aligned to the human genome reference sequence 19 (hg19), and variant annotation and analysis were carried out using Genomize Seq (Genomize, İstanbul, Turkey). In WES analysis, Phenotype-driven filtering was performed using Human Phenotype Ontology (HPO) terms. Core terms such as “seizure” (HP:0001250) and “epileptic encephalopathy” (HP:0200134) were applied to all patients. To further refine variant prioritization, additional HPO terms reflecting each patient’s individual clinical features (e.g., microcephaly, spasticity, hypotonia, movement disorder, structural brain anomaly, facial dysmorphism) were incorporated. This phenotype-centered approach enabled the detection of both well-established epilepsy-associated genes and potential novel genotype–phenotype correlations. Genomize Seq, Franklin, ClinVar, OMIM, MalaCards, PubMed, and segregation analysis were used for variant interpretation. Detected variants were classified as “pathogenic”, “likely pathogenic”, or “variants of uncertain significance (VUS)” according to the ACMG criteria. Segregation analysis was conducted using trio-based strategies, encompassing both trio-based WES and trio-based gene panel. Variants identified in probands were systematically evaluated in both parents to establish inheritance patterns. Visualization and verification of candidate variants were performed using the Integrative Genomics Viewer (IGV, San Diego, CA, USA), ensuring accurate assessment of familial segregation. For the assessment of de novo variants according to the PS2 criterion, maternity and paternity were confirmed before analysis.

## 3. Results

A targeted gene panel was performed in a total of 129 patients with DEE. The cohort included 66 males and 63 females, with a median age at genetic testing of 4.69 years (range: 1 month to 17 years). Forty-nine patients (37.98%) were diagnosed by Trio-analysis.

**Figure 1:**
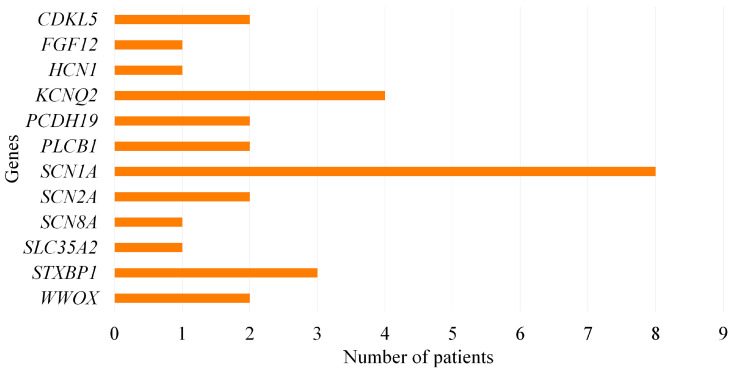
Distribution of pathogenic and likely pathogenic variants among genes.

Pathogenic and likely pathogenic variants were identified in 29 of the 129 patients, resulting in a diagnostic yield of 22.48%. Pathogenic and likely pathogenic variants were detected in *SCN1A* (*n* = 8), *KCNQ2* (*n* = 4), *STXBP1* (*n* = 3), *CDKL5* (*n* = 2), *PCDH19* (*n* = 2), *PLCB1* (*n* = 2), *WWOX* (*n* = 2), *SCN2A* (*n* = 2), *FGF12* (*n* = 1), *HCN1* (*n* = 1), *SCN8A* (*n* = 1), *SLC35A2* (*n* = 1) genes (Figure 1). Among these genes, 12 novel variants were found in 7 genes: *CDKL5*, *HCN1*, *SCN1A*, *SCN2A*, *SCN8A*, *SLC35A2*, and *STXBP1*. *SCN1A* was the most frequently affected gene in the pathogenic/likely pathogenic variants group and was detected in 7 patients with DS. The variant types included 4 missense, 1 nonsense, 1 splice-region, and 1 stop-gained. The genes affected in patients with WS were *WWOX*, *SLC35A2*, *SCN2A*, and *SCN1A*. In the EIDEE patients, *KCNQ2* was the most commonly affected gene, followed by *STXBP1*, *CDKL5*, *FGF12*, *SCN2A*, and *WWOX*. *PCDH19*, *PLCB1*, *SCN8A*, and *HCN1* variations were found in Late Infantile Developmental and Epileptic Encephalopathy (LIDEE) patients.

According to the segregation analysis of available family members, we identified de novo variants in 18 patients (13.95%). A homozygous *WWOX* gene variant was detected in two siblings (case #49–50) from a consanguineous family. We identified two siblings (cases #23–24) with a homozygous variant in *PLCB1* inherited from non-consanguineous, heterozygous parents.

Demographic and clinical data with descriptions of pathogenic and likely pathogenic variants of the cohort are summarized in Table 1.

Variants of uncertain significance (VUS) were found in 9 patients (6.98%). VUS variants were identified in *SPTAN1* (*n* = 2), *CACNA1A* (*n* = 1), *FGF12* (*n* = 1), *GABRA1* (*n* = 1), *HNRNPU* (*n* = 1), *KCNB1* (*n* = 1), *NECAP1* (*n* = 1) and *MDH2* (*n* = 1) genes. Among these genes, 3 novel variants were found in 3 genes: *FGF12*, *NECAP1*, and *SPTAN1*. *SPTAN1* was the most frequently affected gene in the VUS group. The genes affected in patients with WS were *SPTAN1*, *KCNB1*, *HNRNPU*, *FGF12*, and *CACNA1A*. *SPTAN1* and *GABRA1* variations were found in patients with DS. In a patient with EIDEE (case #19), a homozygous *MDH2* variant inherited from a consanguineous family was detected. Likewise, we found a patient (case #20) with a *NECAP1* variant in a homozygous state inherited from a consanguineous family. According to the segregation analysis of available family members, six variants were inherited from either one of the asymptomatic parents. These inherited VUS were retained as VUS, reflecting the potential for incomplete penetrance and variable expressivity in dominant epilepsy-associated genes. Segregation analysis was recommended in extended family members when affected individuals were available. All VUS were subjected to ongoing clinical follow-up and periodic re-evaluation as new functional or literature evidence emerged.

Table 2 summarizes the demographic and clinical data of patients with detected VUS variants.

**Figure 2:**
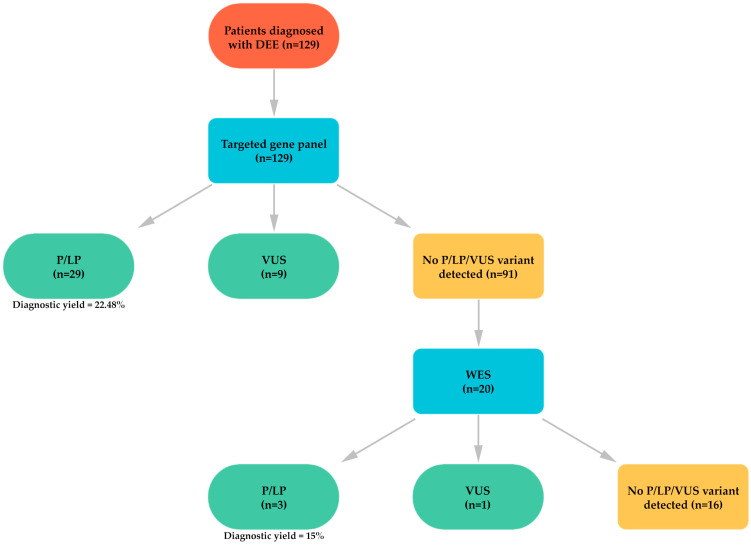
Schematic representation of the study cohort and diagnostic yields of WES and gene panel.

In 91 (70.54%) patients, no pathogenic, likely pathogenic, or VUS explaining the clinical condition was identified through the targeted gene panel. Among these patients, 20 were referred for further evaluation with WES. The variants identified through WES and the phenotypic findings of the patients are presented in Table 3. Figure 2 provides a schematic representation of the study cohort, including the number of patients analyzed by gene panel and WES, together with the diagnostic yield of each approach.

In the WES results, pathogenic and likely pathogenic variants were identified in 3 of the 20 patients, resulting in a diagnostic yield of 15%. Pathogenic and likely pathogenic variants were detected in *TSC1* (*n* = 1), *NALCN* (*n* = 1), and *CTBP1* (*n* = 1) genes. VUS were found in 1 patient (5%). A VUS variant was identified in the *PIEZO2* (*n* = 1) gene. Of the 4 identified distinct OMIM phenotypes, 3 were autosomal dominant, 1 was autosomal recessive. Among the families who underwent family-based trio sequencing, de novo variants were identified in 2 cases (cases #52 and #59), carrier status was observed in one non-consanguineous couple (case #53).

Case #52 was a 1-year-old male with developmental delay and infantile spasms. Electroencephalogram EEG findings revealed hypsarrhythmia, consistent with WS. Renal ultrasonography showed bilateral renal echogenicity and nephrolithiasis. There were no cutaneous features suggestive of Tuberous Sclerosis Complex (TSC), such as hypomelanotic macules or facial angiofibroma. Genetic testing revealed a de novo heterozygous likely pathogenic variant in the *TSC1* gene. No family history of TSC or related disorders was reported. The patient was treated with vigabatrin, which resulted in control of seizures.

Case #53 was a 9-year-old female, born to non-consanguineous parents, who presented with developmental delay and refractory seizures. She was diagnosed with WS. Clinical examination revealed moderate cerebral dysfunction with mild-to-moderate active multifocal epileptic abnormalities on EEG, episodes of respiratory distress, laryngomalacia, and recurrent atelectasis, secundum atrial septal defect, and asymmetric septal hypertrophy, persistent feeding challenges necessitating nutritional support, and scoliosis. Whole-exome sequencing revealed a homozygous likely pathogenic variant in the *NALCN* gene. Parental testing confirmed heterozygosity for the variant in both parents.

The patient, a 1-year-old male, was referred for evaluation due to global developmental delay, hypotonia, and refractory infantile spasms. He was a child of consanguineous parents, with an unremarkable family history. Prenatal and perinatal histories were uneventful. On examination, the patient exhibited severe hypotonia and feeding difficulties requiring nutritional support. Neurological assessment revealed myoclonus and infantile spasms. Dysmorphic features included pectus excavatum. MRI of the brain showed pachygyria, a thin corpus callosum, and a hypoplastic inferior vermis. Additionally, enamel hypoplasia and delayed eruption of teeth were noted. Whole exome sequencing identified a heterozygous de novo likely pathogenic variant in *CTBP1*, consistent with the clinical diagnosis of Hypotonia, Ataxia, Developmental Delay, and Tooth Enamel Defect Syndrome. Parental testing confirmed the absence of the variant in both parents.

A 1-year-old female (case #51) was referred for evaluation due to developmental delay and seizures. The patient had been followed with EIDEE. On clinical examination, she exhibited microcephaly, abnormal motor development, and dystonic movements. Neuroimaging revealed a thin corpus callosum, enlarged lateral ventricles, and polymicrogyria. Additionally, EEG findings showed frequent spike, polyspike, and slow-wave discharges localized to the left central and right temporal regions. The patient demonstrated bicycling movements of both feet, consistent with dystonia. She had frequent hospitalizations due to respiratory distress. Genetic testing revealed a heterozygous VUS variant in the *PIEZO2* gene, which is associated with Marden–Walker syndrome.

## 4. Discussion

Our single-center retrospective cohort study provides a comprehensive analysis of genetic findings in a cohort of 129 patients with DEE, revealing insights about diagnostic yield, gene-specific findings, and the role of segregation analysis.

In this study, pathogenic and likely pathogenic variants were identified in 29 of the 129 patients, resulting in a diagnostic yield of 22.48%. This yield is consistent with previous studies with targeted gene panels including 5-580 genes that reported diagnostic yields, which range from 18 to 46.2% [3,10,11,12,13,14,15,16,17]. These studies are summarized in Table 4. The variability of the diagnostic yields may depend on factors such as cohort size, gene numbers, and patient selection criteria. Our diagnostic yield underscores the effectiveness of the targeted gene panel in identifying the genetic causes of DEE and also underscores the validity of our findings.

In the present study, eight heterozygous *SCN1A* variants were identified in patients presenting with developmental and epileptic encephalopathies, predominantly DS and, in one case, WS. The observed spectrum included protein-truncating variants (frameshift, nonsense, and stop-gained), missense substitutions, and a splice-site alteration. All variants were confirmed as de novo and classified as pathogenic. *SCN1A*-related disorders encompass a broad phenotypic spectrum, ranging from mild presentations, such as simple febrile seizures and generalized epilepsy with febrile seizures plus (GEFS+), to severe conditions like DS and intractable childhood epilepsy with generalized tonic–clonic seizures (ICE-GTC) [4,18,19]. *SCN1A* pathogenic variants account for approximately 70–80% of classical DS cases, underscoring their central role in this severe developmental and epileptic encephalopathy. In *SCN1A*, nearly 45–55% of DS cases are explained by pathogenic missense substitutions, while the remaining cases arise from protein-truncating variants (PTVs), copy number changes, or other mutation types. PTVs typically produce haploinsufficiency, resulting in a complete loss of function (LOF). In contrast, missense variants can exert a range of effects on channel activity: those that result in a complete loss of function altogether are more often linked to DS, whereas variants that only partially reduce channel activity are frequently associated with the milder phenotypes [7]. In this study, two patients with DS carried truncating variants: a stop-gained at p.S683Ter (case #29), and a nonsense variant at p.W1347Ter (case #30). Four patients carried missense variants: p.C257R, p.G1480C, p.Q554L, and p.N139S. Missense variants show location-dependent effects, with those in critical regions—voltage-sensing (S4) and pore-forming segments (S5-S6)—typically causing earlier onset and severe DS [7]. Accordingly, p.C257R (Domain I) (case #26), p.G1480C (Domain III) (case #28), and p.Q554L (case #31) were all associated with DS, reflecting functional disruption. In contrast, p.N139S (Domain I) (case #32) in the N-terminal region, although generally linked to milder phenotypes [20], presented as DS and was de novo. Case #27 carried a splice donor variant (c.2589+3A>T), predicted to alter splicing and result in loss of function, consistent with the observed DS phenotype This variant profile likely explains the predominance of DS in our cohort, as such variants are known to cause severe haploinsufficiency of the *SCN1A*-encoded Nav1.1 sodium channel, leading to early-onset febrile and afebrile seizures, multiple seizure types, developmental delay, and characteristic EEG abnormalities. Notably, one patient (case #25) presented with WS, carrying a frameshift variant (c.1013delA, p.N338fs), consistent with prior reports documenting *SCN1A*-associated WS cases, while it is rarely linked to *SCN1A* [21,22,23,24]. This underscores the clinical heterogeneity of truncating *SCN1A* variants and adds to the small but growing number of reports linking *SCN1A* to WS. These findings, together with the literature, underscore the importance of careful genotype–phenotype correlation and consideration of *SCN1A* testing even in atypical early-onset epilepsies.

In our cohort, genetic analysis revealed disease-causing variants associated with WS not only in *SCN1A*, but also in *SCN2A*, *SLC35A2*, and *WWOX*, each associated with distinct clinical patterns. *SCN2A* missense variant (p.E1211K) (case #33) was associated primarily with behavioral abnormalities, multifocal epileptiform activity on a disorganized EEG background, and early-onset seizures, supporting literature indicating that this variant changes the functional property of the sodium channel and contributes to severe phenotypes with neuropsychiatric manifestations such as autistic features [25]. Patient harboring *SLC35A2* (p.S258F) variant (case #40) exhibited multifocal epileptiform EEG patterns and hypsarrhythmia, reflecting the severe early-onset epileptic phenotypes described in congenital disorders of glycosylation [26]. Additionally, *WWOX* missense variant (p.L239R) identified in patient (case #49) from a consanguineous family was linked to hypsarrhythmic EEG patterns and multifocal seizures consistent with previous reports emphasizing the gene’s role in early-onset epilepsy and poor neurodevelopmental outcomes and linking to *WWOX*-related epileptic encephalopathy (DEE28 or WOREE syndrome) [27]. In this study, WS was observed predominantly in male patients, consistent with previous reports suggesting a slight male predominance in early-onset epileptic encephalopathies [28]. Altogether, WS demonstrated marked genetic heterogeneity, with disease-causing variants identified in *SCN1A*, *SCN2A*, *SLC35A2*, and *WWOX*, each conferring distinctive clinical and electroencephalographic patterns. *SCN1A* frameshift variants were linked to severe, early-onset phenotypes with structural brain abnormalities, whereas *SCN2A* missense variants were associated with early-onset seizures with behavioral disturbances. The *SLC35A2* variant reflected the contribution of congenital disorders of glycosylation and supports the metabolic etiology of WS. In contrast, *WWOX* variants were characterized by hypsarrhythmia, multifocal seizures, and poor neurodevelopmental outcomes consistent with WOREE syndrome.

Patients with EIDEE in our cohort exhibited disease-causing variants in *CDKL5*, *KCNQ2*, *SCN2A*, *FGF12*, *STXBP1*, and *WWOX*, with a predominance of de novo events. *CDKL5* variants, including a frameshift (p.Q298VfsTer46) and a missense (p.K412Q), were associated with early-onset disorganized EEG patterns and drug-resistant seizures, consistent with the literature highlighting *CDKL5* as a major contributor to severe EIDEE phenotypes. Notably, frameshift variants tend to result in more severe clinical outcomes compared to missense variants, reflecting their stronger disruptive effect on protein function [29]. *KCNQ2*-related EIDEE (cases #15–18) was caused by heterozygous missense variants located in the pore domain (p.T274M, p.A306V) or the S4 voltage-sensing domain (p.S195F, p.R207W). Variants in the pore domain produce loss of function and disrupted potassium conductance, leading to early-onset seizures, and S4 domain variants alter voltage-dependent gating, producing focal seizures due to localized neuronal hyperexcitability [30]. EEG recordings revealed focal epileptiform discharges in cases #17–18. Clinically, all patients exhibited early-onset seizures, and one (case #18) additionally demonstrated autism spectrum features, highlighting the broader neurodevelopmental impact of S4 dysfunction. In our cohort, the patient carrying the *SCN2A* (p.I894S) missense variant presented with early-onset epileptic encephalopathy characterized by multifocal tonic and focal seizures with neonatal onset, consistent with previous reports linking Domain II S5–S6 pore-region variants to severe epilepsy. Although p.I894S has not been functionally characterized, its location in the Domain II pore-forming region suggests a gain-of-function effect, similar to neighboring residues reported in the literature (e.g., p.A890T, p.I892T, p.V896M). This correlates with the patient’s early seizure onset and refractory epileptic activity. Our observation reinforces the established genotype–phenotype correlation in *SCN2A*-related disorders, highlighting that missense variants in the Domain II pore region tend to confer severe epileptic phenotypes, whereas variants located elsewhere or causing loss-of-function may present with milder or primarily neurodevelopmental manifestations. Gain-of-function variants, which result in greater NaV1.2 activity than normal, are associated with neonatal and infantile-onset epilepsy. In contrast, loss-of-function variants are associated with later-onset epilepsy with more prominent neurodevelopmental delay as well as autism spectrum disorder [31]. The *FGF12* missense variant (p.R114H, case #6) presented with focal epileptiform anomalies, thin corpus callosum, and decreased white matter on MRI. This phenotype aligns with previous reports, where *FGF12* variants were shown to cause Developmental and epileptic encephalopathy 47, frequently manifesting as EIDEE. Patients carrying *FGF12* variants were more frequently observed to have focal epileptiform discharges and cerebellar atrophy [32]. Thus, our case further supports the role of *FGF12* in early-onset epileptic encephalopathy, reinforcing the association with focal EEG abnormalities and structural brain changes.

In our cohort, three patients harbored heterozygous pathogenic or likely pathogenic *STXBP1* variants, all presenting with EIDEE but exhibiting variable clinical manifestations and EEG features according to the mutation type. The patient with a nonsense variant (c.547delC) demonstrated focal epileptiform discharges along with hearing loss and autism-like behavior, suggesting that truncating variants may contribute to a broader neurodevelopmental phenotype beyond epilepsy. The splice-site variant (c.664-2A>G) was associated with multifocal generalized epileptiform activity, consistent with severe epileptic phenotypes often linked to loss-of-function variants, while the missense variant (p.G568R), occurring de novo, again presented with focal discharges on a disorganized background. These observations suggest a possible genotype-EEG correlation, where nonsense and missense variants more often present with focal discharges, whereas splice-site variants tend to produce multifocal generalized activity. This spectrum highlights the broad phenotypic heterogeneity of *STXBP1*-related encephalopathies, and although focal epileptiform activity is not specific to *STXBP1*, it remains one of the most frequently reported EEG findings. Our findings support prior reports that EEG patterns may partly reflect the functional consequences of different variant types [33]. A homozygous missense variant in *WWOX* (c.716T>G, p.L239R) was identified in an infant presenting with hypsarrhythmia and multifocal epileptiform anomalies on a disorganized EEG background, consistent with EIDEE. While most previously reported cases involve truncating or splice-site variants leading to loss of function, emerging evidence indicates that certain missense variants may also lead to a somewhat milder course in terms of overall prognosis; nevertheless, in terms of epileptology, patients with missense variants still present with very early onset and multiple seizure types, frequently focal and spasms [34].

In our cohort, six patients were diagnosed with LIDEE, harboring pathogenic or likely pathogenic variants in *HCN1*, *PCDH19*, *PLCB1*, and *SCN8A*. To our knowledge, genotype–phenotype studies specifically focusing on LIDEE are relatively scarce in the literature. The *HCN1* variant (c.365A>G, p.K122R) occurred de novo in a female patient presenting with myoclonic seizures and focal epileptiform anomalies on EEG, consistent with prior reports highlighting that LIDEE represents a clinically and electrographically distinct DEE syndrome including common seizure types as myoclonic–tonic, tonic, epileptic spasms, and myoclonic seizures, often accompanied by disorganized EEG background with low-voltage and multifocal epileptiform discharges [35]. Two female patients carried the same *PCDH19* frameshift variant (c.1091dup, p.Y366Lfs*10), presenting with intellectual disability, scoliosis, dermatological symptoms, and, in one, menstrual irregularity. These findings align with the known phenotype spectrum of *PCDH19*-related DEE, in which heterozygous females exhibit early-onset, drug-resistant seizures, neurodevelopmental delay, and systemic features, while male carriers are usually unaffected [36]. Two siblings (one female, one male) were homozygous for the *PLCB1* missense variant (c.1945A>G, p.R649G), both presenting with malignant migrating focal seizures and multifocal epileptiform EEG abnormalities. The homozygous state and severe seizure phenotype suggest a recessive inheritance pattern, consistent with prior *PLCB1*-related DEE [37]. Finally, a male patient with a *SCN8A* missense variant (c.5246A>G, p.Y1749C) exhibited psychomotor delay and ischemic changes on MRI, highlighting the contribution of *SCN8A* variants to LIDEE phenotypes characterized by developmental delay and multifocal epileptiform activity [38]. This variant affects the DIVS6 segment of the channel, and gain-of-function variants in this region have been reported to cause severe, early-onset epilepsy, whereas loss-of-function variants are typically linked to later-onset phenotypes [39]. Functional analyses are required to determine whether this variant results in a gain- or loss-of-function effect and thereby to clarify its precise pathogenic mechanism. Collectively, these findings underscore the genetic heterogeneity of LIDEE and the correlation between variant type and clinical severity, with frameshift and homozygous missense variants generally associated with more profound neurodevelopmental and epileptic manifestations. The limited number of published genotype–phenotype studies in LIDEE emphasizes the need for further systematic investigations to better characterize this clinically heterogeneous condition.

In addition to pathogenic and likely pathogenic variants, VUS were detected in *CACNA1A*, *FGF12*, *GABRA1*, *HNRNPU*, *KCNB1*, and *SPTAN1* genes. VUS were found in 6.98% of patients. This rate is compatible with the VUS rate in previous studies conducted with panels including a few genes [12], but is lower than the previously reported VUS rate detected with panels including more than 100 genes [3,15,16]. It has been reported that large panels, including hundreds of genes, resulted in a higher VUS rate compared to gene panels including a few genes [12]. The majority of our patients underwent trio-analysis. Therefore, the classification of the initial VUS variant could have been classified as likely benign/benign or likely pathogenic/pathogenic according to the parent results.

The majority of our patients underwent trio-analysis, which enabled more precise variant classification. Therefore, the initial VUS could have been reclassified as likely benign/benign or likely pathogenic/pathogenic according to the parental results. Nevertheless, inherited VUS, particularly those from apparently unaffected parents, present additional challenges for clinical interpretation. Incomplete penetrance and variable expressivity can obscure genotype–phenotype correlations, complicating risk assessment and genetic counseling. Therefore, careful evaluation of these inherited VUS is essential for guiding family planning decisions and follow-up strategies, and longitudinal clinical monitoring, together with functional studies, is recommended to clarify pathogenicity and improve the accuracy of risk prediction.

In parallel with VUS assessment, the segregation analysis revealed de novo variants in 13.95% of patients. Previous studies have indicated that between 30% and 50% of DEEs are associated with a pathogenic variation, and the majority of these occur de novo [40]. This result supports the notion that many genetic variants in DEE arise spontaneously rather than being inherited.

Our study expands the genotype–phenotype correlations in WS by identifying pathogenic or likely pathogenic variants in *TSC1*, *NALCN*, and *CTBP1* using WES. Detailed clinical descriptions of patients were provided, highlighting their distinctive or novel features that broaden the known phenotypic spectrum of the associated syndromes. By integrating comprehensive clinical characterization with molecular findings, insights into broader phenotypic spectra are provided, and clinical monitoring is informed.

Case #52 was diagnosed with WS. Genetic analysis identified a de novo heterozygous likely pathogenic variant in the *TSC1* gene. TSC is a recognized genetic cause of WS [41]. Renal involvement is common in TSC; however, the presence of nephrolithiasis (kidney stones) is not commonly reported in TSC-related renal pathology [42]. The absence of skin lesions can pose a diagnostic challenge. It has been reported that identifying a pathogenic variation in *TSC1* or *TSC2* is enough to confirm the diagnosis [43]. This case, involving a *TSC1* variant with WS and renal involvement but without skin lesions, highlights the necessity of continuous medical surveillance. Even in the absence of overt cutaneous features, the potential for disease progression in other organ systems necessitates careful and prolonged clinical monitoring.

A homozygous likely pathogenic variant was detected in the *NALCN* gene in a female with WS (case #53). Variants in *NALCN* (sodium leak channel, non-selective) have been implicated in Infantile Hypotonia with Psychomotor Retardation and Characteristic Facies-1 (IHPRF1), an autosomal recessive condition characterized by moderate-severe hypotonia, developmental delay, and distinctive facial features. Our patient shares several overlapping features with IHPRF1 cases but also exhibits rare and unique findings [44]. Secundum atrial septal defect and asymmetric septal hypertrophy have been infrequently reported in NALCN-related disorders, suggesting novel phenotypic features. However, some reports have noted cardiac involvement, such as atrial septal defect and tricuspid regurgitation in affected individuals who exhibited a range of severe symptoms [45,46]. Cardiac anomalies and their occurrence in some patients highlight the importance of comprehensive cardiac evaluations in individuals with NALCN variants. While seizures are reported in IHPRF1, to date, there are no reported cases in the literature with WS, making this the first documented case linking these conditions. This case expands the clinical spectrum of *NALCN*-related disorders and highlights the need for further investigation into the potential epileptogenic role of *NALCN* variants.

WES identified a heterozygous de novo likely pathogenic variant in *CTBP1* in a patient (case #59). On examination, the patient exhibited clinical features consistent with *CTBP1*-related hypotonia, ataxia, developmental delay, and tooth enamel defect syndrome (HADDTS). HADDTS is an extremely rare disorder. To date, there have been only 15 cases described in the literature [47]. Our patient shares a unique feature with a Chinese pedigree case: the presence of pectus excavatum, a condition that has not been reported in the other 14 known cases of HADDTS. Moreover, WS, which is typically associated with DEEs, has not been previously reported in HADDTS patients. We describe the first such case of HADDTS with WS, further expanding the spectrum of neurological symptoms that can be associated with this condition. In the literature, non-refractory myoclonic seizures beginning at the age of five have been described in a 24-year-old male [48]. The variability in clinical presentation and the emerging recognition of rare features such as pectus excavatum and WS highlight the need for clinicians to be aware of the broader phenotypic spectrum of HADDTS.

In the current study WES diagnostic yield of 15% is lower than previous studies, showing WES diagnostic rates between 25% and 45% [49,50]. One reason could be the limited number of patients who underwent WES. Among the patients for whom a targeted gene panel failed to establish a definitive genetic cause, only 20 patients were able to receive WES due to resource constraints. This small sample size may have reduced the likelihood of identifying pathogenic variants, leading to a lower overall diagnostic yield.

Targeted gene panels have been widely used in DEE diagnosis, but their limitations in detecting novel variants reduce their effectiveness compared to WES. Several studies have demonstrated that WES significantly increases diagnostic yield, particularly in cases where rare or previously unreported variants are involved [51]. Our findings reinforce this, as one of the identified variants (*PIEZO2*) is novel and would likely have been missed using a targeted panel approach. A heterozygous VUS variant in the *PIEZO2* gene was detected in a patient diagnosed with EIDEE (case #51). The patient exhibited clinical features consistent with Marden–Walker syndrome. The syndrome often manifests various congenital contractures, psychomotor and growth retardation, immobility of the face, and diminished muscle mass. Additionally, problems of the heart, kidneys, and brain have been reported [52,53,54], but epilepsy is rare. Notably, epilepsy is not a common feature of Marden–Walker syndrome, but the presence of abnormal EEG findings in this case suggests a possible extension of the neurological phenotype associated with *PIEZO2* variants.

## 5. Conclusions

This study highlights the genetic heterogeneity and phenotypic variability of DEE and the utility of next-generation sequencing, including WES and targeted gene panels, in elucidating the genetic causes of DEE. The present study is a retrospective single-center design which have some limitations. First, a retrospective single-center design may restrict access to the clinical data and generalizability of our findings to broader populations. The second limitation is that the relatively small number of patients who underwent WES was primarily due to financial constraints and limited insurance coverage. Insurance coverage for WES is often limited or non-existent, leading to outright denials or inadequate reimbursement. Consequently, many patients either forgo testing or are excluded from studies due to these financial and coverage barriers. These limitations underscore the need for policy reforms to improve access to genetic testing and facilitate broader participation in genomic research.

While targeted panels offer a faster turnaround time, WES provides a broader diagnostic scope, identifying both known and novel variants. Our findings emphasize that WES can be used as a first-tier diagnostic tool in epilepsy syndromes and DEE. The observed genetic and phenotypic heterogeneity underscores the need for a genotype-first approach, multidisciplinary evaluation, and long-term follow-up to optimize diagnosis and personalized patient care.

## Figures and Tables

**Table 1:** Demographic and clinical data of patients with pathogenic and likely pathogenic variants.

Gene	Variant	Reported ACMG Classification	Case ID	Sex/Age at the Study (Years/Months) *	Clinical Features/Epileptic Syndrome	Novel/ Previously Reported Variant	Pattern of Inheritance (De Novo/Maternal/ Paternal)	Consanguinity
*CDKL5*	Heterozygous Frameshift chrX:18616648 NM_001323289.2 c.892_911delinsG p.Q298VfsTer46	P (PVS1, PS2, PM2)	3	F/10	Epileptiform anomaly on a disorganized background in EEG/EIDEE	Novel	de novo	N/A
	Heterozygous Missense chrX:18622278 NM_001323289.2 c.1234A>C p.K412Q	LP (PS2, PM2)	4	M/5 months	Seizures/EIDEE	Novel	de novo	N/A
*FGF12*	Heterozygous Missense chr3:192053223 NM_004113.6 c.341G>A p.R114H	P (PS2, PS3, PM2, PP3, PP5)	6	F/6	Thin corpus callosum and decreased white matter volume in MRI, focal epileptiform anomaly on severely disorganized background in EEG/EIDEE	ClinVar 2584544	de novo	No
*HCN1*	Heterozygous Missense chr5:45695831 NM_021072.4 c.365A>G p.K122R	LP (PS2, PM2, PP2)	12	F/10 months	Myoclonic seizures, focal epileptiform anomaly in EEG/LIDEE	Novel	de novo	No
*KCNQ2*	Heterozygous Missense chr20:62071057 NM_172107.4 c.821C>T p.T274M	P (PS2, PS3, PM1, PM2, PM5, PP2, PP3)	15	F/1	Seizures/EIDEE	ClinVar 167208	de novo	N/A
	Heterozygous Missense chr20:62070961 NM_172107.4 c.917C>T p.A306V	P (PM1, PM2, PM5, PP2, PP3, PP5)	16	M/3 months	Disorganized background in EEG/EIDEE	ClinVar 219235	N/A	N/A
	Heterozygous Missense chr20:62076118 NM_172107.4 c.584C>T p.S195F	P (PM1, PM2, PM5, PP2, PP3, PP5)	17	M/2 months	Focal epileptiform anomaly on a disorganized background in EEG/EIDEE	ClinVar 590184	N/A	N/A
	Heterozygous Missense chr20:62076083 NM_172107.4 c.619C>T p.R207W	P (PS2, PS3, PM1, PM2, PM5, PP2, PP3)	18	M/5	Autism, focal epileptiform anomaly in EEG/EIDEE	ClinVar 7386	de novo	N/A
*PCDH19*	Heterozygous Frameshift chrX:99662504 NM_001184880.2 c.1091dup p.Y366Lfs*10	P (PVS1, PS4, PM2)	21	F/16	Intellectual disability, scoliosis, dermatological symptoms/LIDEE	ClinVar 206353	N/A	N/A
	Heterozygous Frameshift chrX:99662504 NM_001184880.2 c.1091dup p.Y366Lfs*10	P (PVS1, PS4, PM2)	22	F/17	Menstrual irregularity, dermatological symptoms/LIDEE	ClinVar 206353	N/A	N/A
*PLCB1*	Homozygous Missense chr20:8713941 NM_015192.4 c.1945A>G p.R649G	LP (PM2, PP1, PP2, PP3, PP4)	23	F/6	Multifocal epileptiform anomaly in EEG, malignant migrating focal seizures/LIDEE	SNP rs1219807693	Carrier parents	No
	Homozygous Missense chr20:8713941 NM_015192.4 c.1945A>G p.R649G	LP (PM2, PP1, PP2, PP3, PP4)	24	M/3	Multifocal epileptiform anomaly in EEG, malignant migrating focal seizures/LIDEE	SNP rs1219807693	Carrier parents	No
*SCN1A*	Heterozygous Frameshift chr2:166905410 NM_001165963.4 c.1013delA p.N338fs	P (PVS1, PS2, PM2)	25	M/7 months	Thin corpus callosum in MRI, focal epileptiform anomaly in EEG/WS	Novel	de novo	N/A
	Heterozygous Missense chr2:166908424 NM_001165963.4 c.769T>C p.C257R	P (PS2, PS4, PM1, PM2, PP2, PP3)	26	F/4	Multifocal generalized epileptiform anomaly in EEG/DS	ClinVar 189934	de novo	No
	Heterozygous Splice region chr2:166895930 NM_001165963.4 c.2589+3A>T	P (PS2, PS3, PM2, PP3)	27	F/9 months	Focal epileptiform anomaly in EEG/DS	ClinVar 189938	de novo	N/A
	Heterozygous Missense chr2:166854586 NM_001165963.4 c.4438G>T p.G1480C	P (PS2, PM1, PM2, PM5, PP2, PP3)	28	F/3	Focal epileptiform anomaly in EEG/DS	ClinVar 206830	de novo	N/A
	Heterozygous Stop gained chr2:166898897 NM_001165963.4 c.2048C>A p.S683Ter	P (PVS1, PS2, PS4, PM2)	29	M/10	Seizures/DS	ClinVar 2431708	de novo	Yes
	Heterozygous Nonsense chr2:166859193 NM_001165963.4 c.4040G>A p.W1347Ter	P (PVS1, PS2, PS4, PM2)	30	F/10 months	Focal epileptiform anomaly on a disorganized background in EEG/DS	ClinVar 1458263	de novo	Yes
	Heterozygous Missense chr2:166901554 NM_001165963.4 c.1661A>T p.Q554L	P (PS2, PM2, PM5, PP2, PP3)	31	F/6	Intellectual disability/DS	Novel	de novo	No
	Heterozygous Missense chr2:166859091 NM_001165963.4 c.4175A>G p.N139S	P (PS2, PM1, PM2, PP2, PP3)	32	F/16	Seizures/DS	Novel	de novo	N/A
*SCN2A*	Heterozygous Missense chr2:166223837 NM_001040142.2 c.3631G>A p.E1211K	P (PS2, PM2, PP2, PP3, PP5)	33	M/8	Severe neuromotor delay, behavioral disorder, multifocal epileptiform anomaly on a disorganized background in EEG/WS	ClinVar 29886	de novo	N/A
	Heterozygous Missense chr2:166201183 NM_001040142.2 c.2681T>G p.I894S	P (PS2, PM1, PM2, PP2, PP3)	34	M/4 months	Seizures/EIDEE	Novel	de novo	N/A
*SCN8A*	Heterozygous Missense chr12:52200516 NM_001330260.2 c.5246A>G p.Y1749C	LP (PM2, PP2, PP3)	36	M/16	Psychomotor delay, ischemia sign in MRI/LIDEE	Novel	N/A	No
*SLC35A2*	Heterozygous Missense chrX:48762497 NM_005660.3 c.773C>T p.S258F	LP (PS2, PM2)	40	M/6	Generalized epileptiform anomaly in EEG/WS	Novel	de novo	No
*STXBP1*	Heterozygous Nonsense chr9:130425598 NM_001032221.6 c.547delC p.Leu183Ter	LP (PVS1, PM2)	44	M/1	Hearing loss, autism-like behavior, focal epileptiform anomaly in EEG/EIDEE	Novel	N/A	No
	Heterozygous Splice acceptor chr9:130428443 NM_001032221.6 c.664-2A>G	P (PVS1, PM2, PP3)	45	F/1	Multifocal generalized epileptiform anomaly in EEG/EIDEE	Novel	N/A	No
	Heterozygous Missense chr9:130444839 NM_001032221.6 c.1702G>A p.G568R	P (PS1, PS2, PM1, PM2, PP2, PP3)	47	F/16	Focal epileptiform anomaly on a disorganized background in EEG/EIDEE	Novel	de novo	N/A
*WWOX*	Homozygous Missense chr16:78458877 NM_016373.4 c.716T>G p.L239R	P (PM2, PM3, PP3, PP5)	49	M/3 months	Multifocal epileptiform anomaly in EEG/WS	ClinVar 871669	Carrier parents	Yes
	Homozygous Missense chr16:78458877 NM_016373.4 c.716T>G p.L239R	P (PM2, PM3, PP3, PP5)	50	F/11 months	Hypsarrhythmia and multifocal epileptiform anomaly on a disorganized background in EEG/EIDEE	ClinVar 871669	Carrier parents	Yes

Abbreviations: M, Male; F, Female; EIDEE, Early Infantile Developmental and Epileptic Encephalopathy; LIDEE, Late Infantile Developmental and Epileptic Encephalopathy; WS, West syndrome; DS, Dravet syndrome; P, Pathogenic; LP, Likely Pathogenic; N/A, Information not available; EEG, Electroencephalogram; MRI, Magnetic Resonance Imaging. * “Age at study” refers to the age at examination.

**Table 2:** Demographic and clinical data of patients with variants of uncertain significance.

Genes	Variant	Reported ACMG Classification	Case ID	Sex/Age at the Study (Years/Month)	Clinical Features/Epileptic Syndrome	Novel/ Previously Reported Variant	Pattern of Inheritance (De Novo/Maternal/ Paternal)	Consanguinity
*CACNA1A*	Heterozygous Missense chr19:13409974 NM_001127222.2 c.2473G>A p.V825M	VUS (PM2, PP2)	1	M/5 months	Learning disability, generalized epileptiform anomaly on a disorganized background in EEG/WS	ClinVar 1485168	Paternal	Yes
*FGF12*	Heterozygous Splice region chr3:191888452 NM_004113.6 c.415-9_415-7delTTCinsCTT	VUS (PM2)	5	M/2	Microcephaly, thin corpus callosum, and decreased white matter volume in MRI, focal epileptiform anomaly in EEG/WS	Novel	Paternal	No
*GABRA1*	Heterozygous Missense chr5:161277883 NM_001127644.2 c.67G>A p.G23R	VUS (PM2, PP2)	7	M/2	Seizures/DS	ClinVar 2923865	Paternal	N/A
*HNRNPU*	Heterozygous Missense chr1:245021533 NM_031844.3 c.1274G>C p.G425A	VUS (PM2, PP3)	13	M/6 months	Microcephaly, lobar holoprosencephaly, dysgenesis of corpus callosum, and decreased white matter volume in MRI, generalized focal epileptiform anomaly in EEG/WS	ClinVar 696822	Maternal	No
*KCNB1*	Heterozygous Missense chr20:48099013 NM_004975.4 c.5C>A p.P2Q	VUS (PM2, PP2, BP6)	14	M/1	Developmental delay, thin corpus callosum in MRI/WS	ClinVar 1918447	N/A	No
*MDH2*	Homozygous Missense chr7:75692842 NM_005918.4 c.565C>A p.P189A	VUS (PM2)	19	M/9	Intellectual disability/EIDEE	SNP rs782733818	Carrier parents	Yes
*NECAP1*	Homozygous Initiator codon chr12:8234886 NM_015509.4 c.2T>A p.M1?	VUS (PM2)	20	M/3	Thin corpus callosum in MRI/LIDEE	Novel	Carrier parents	Yes
*SPTAN1*	Heterozygous Missense chr9:131343295 NM_001130438.3 c.1418A>T p.Y473F	VUS (PM2, PP2)	42	M/2	Autism/DS	Novel	Maternal	N/A
	Heterozygous Missense chr9:131341992 NM_001130438.3 c.1298A>G p.Y433C	VUS (PM2)	43	M/9 months	Seizures/WS	ClinVar 1716606	Paternal	N/A

**Table 3:** Whole exome sequencing (WES) findings for 4 patients.

Genes	Variant	Reported ACMG Classification	Case ID	Sex/Age at the Study (Years)	Clinical Features/Epileptic Syndrome	OMIM Phenotype (MIM#)	Pattern of Inheritance (De Novo/Maternal/ Paternal)	Consanguinity
*PIEZO2*	Heterozygous Missense chr18:10696484 NM_001378183.1 c.6542C>T p.P2181L	VUS (PM2, PP2)	51	F/1	Microcephaly, respiratory distress, thin corpus callosum, enlarged lateral ventricles, polymicrogyria, dystonia, frequent spike, polyspike, and slow-wave discharges in the left central and right temporal regions, accompanying bicycling movements of both feet in EEG/EIDEE	Marden–Walker Syndrome (248700), AD	N/A	N/A
*TSC1*	Heterozygous Missense chr9:135787758 NM_000368.5 c.824A>G p.Y275C	LP (PS2, PM2, PP3)	52	M/1	Bilateral renal echogenicity, nephrolithiasis, hypsarrhythmia in EEG/WS	Tuberous sclerosis-1 (191100), AD	de novo	No
*NALCN*	Homozygous Splice donor chr13:101728223 NM_052867.4 c.3954+1G>A	LP (PVS1, PM2)	53	F/9	Cerebral palsy, respiratory distress, feeding difficulties, moderate cerebral dysfunction accompanied by mild-to-moderate active multifocal epileptic abnormalities in EEG, scoliosis, laryngomalacia, atelectasis, secundum atrial septal defect, asymmetric septal hypertrophy/WS	Hypotonia, infantile, with psychomotor retardation and characteristic facies-1 (615419), AR	Carrier parents	No
*CTBP1*	Heterozygous Missense chr4:1207317 NM_001012614.2 c.970T>G p.S324A	LP (PS2, PM2, PP2)	59	M/1	Hypotonia, infantile spasms, feeding difficulties, tooth enamel disease, myoclonus, pectus excavatum, pachygyria, thin corpus callosum, hypoplastic inferior vermis/WS	Hypotonia, ataxia, developmental delay, and tooth enamel defect syndrome (617915), AD	de novo	Yes

AD, Autosomal Dominant; AR, Autosomal Recessive.

**Table 4:** Similar studies using targeted gene panels for DEE.

Study	Number of Patients	Number of Genes	Yield (%)	Frequently Affected Genes
Ream et al. (2014) [11]	25	40–53	46.2	*PCDH19*, *SCN1A*, *SLC2A1*, *SPTAN1*, *SLC9A6*, *EFHC*
Møller et al. (2016) [12]	216	46	23	*SCN1A*, *CDKL5*, *GABRA1*, *GABRB3*, *KCNQ2*, *SCN2A*, *SCN8A*, *SLC2A1*, *STXBP1*
Butler et al. (2017) [13]	339	110	18	*TSC2*, *SCN1A*, *KCNQ2*, *CDKL5*, *SCN2A*, *SCN8A*, *SCN1B*, *STXBP1*, *TPP1*, *PCDH19*, *CACNA1A*, *GABRA1*, *GRIN2A*, *SLC2A1*
Rim et al. (2018) [14]	74	172	37.8	*SXTBP1*, *CDKL5*, *KCNQ2*, *SCN1A*, *SYNGAP1*, *GNAO1*, *KCNT1*
Atli et al. (2020) [15]	80	110	36.25	*TSC2*, *TSC1*, *KCNQ2*, *AMT*, *CACNA1H*, *KCNT1*, *SCN1A*, *GRIN2A*, *CNTNAP2*, *GLDC*, *MECP2*, *ASAH1*
Hoelz et al. (2020) [16]	91	5–434	18	*SCN1A*, *TSC1*, *SCN8A*, *SYNGAP1*, *CPT2*, *KCNB1*, *PCDH19*, *KCNQ2*, *CHD2*, *CACNA1A*, *STXBP1*
Lee et al. (2020) [17]	116	40	34.5	*SCN1A*, *PRRT2*, *ARX*, *SCN2A*, *KCNQ2*, *PCDH19*, *STXBP1*, *DEPDC5*, *SCN8A*
Essajee et al. (2022) [3]	41	308	43.9	*SCN1A*, *KANSL1*, *KCNQ2*, *CDKL5*, *IQSEC2*, *MC1A*, *STXBP1*
Ben Said et al. (2024) [10]	40	116	30	*SCN1A*, *CHD2*, *CDKL5*, *SZT2*, *KCNT1*, *GNAO1*, *PCDH19*, *GRIN2A*, *MECP2*, *SYNGAP1*
Present study (2024)	129	55	22.48	*SCN1A*, *KCNQ2*, *STXBP1*, *CDKL5*, *PCDH19*, *PLCB1*, *SCN2A*, *WWOX*

## Data Availability

The data that support the findings of this study are available from the corresponding author upon reasonable request.
